# Risks Associated With Undiagnosed ADHD and/or Autism: A Mixed-Method Systematic Review

**DOI:** 10.1177/10870547231176862

**Published:** 2023-06-21

**Authors:** Blandine French, David Daley, Madeleine Groom, Sarah Cassidy

**Affiliations:** 1 University of Nottingham, UK; 2 Nottingham Trent University, UK

**Keywords:** ADHD, autism, ASD, systematic review, undiagnosed, undetected

## Abstract

**Background:**

The two most prevalent neurodevelopmental disorders—Attention Deficit Hyperactivity Disorder (ADHD) and Autism (ASD)—(ASD/ADHD) strongly impact individuals’ functions. This is worsened when individuals are undiagnosed and risks such as increased imprisonments, depression or drug misuse are often observed. This systematic review synthesizes the risks associated with late/undiagnosed ASD/ADHD.

**Methods:**

Four databases were searched (Medline, Scopus, PsychInfor, and Embase). Published studies exploring the impact of undiagnosed ASD/ADHD were included. Exclusion criteria included, lack of diagnosis status, studies not solely on ASD or ADHD, gray literature and studies not in English. The findings were summarize through a narrative synthesis.

**Results:**

Seventeen studies were identified, 14 on ADHD and three on ASD. The narrative synthesis identified three main themes: (1) Health, (2) Offending behavior, and (3) Day-to-day impact. The risks highlighted a significant impact on mental wellbeing and social interactions, higher risks of substance abuse, accidents and offending behavior as well as lower levels of income and education.

**Discussion:**

The findings suggest that undiagnosed ASD/ADHD is linked to many risks and negative outcomes affecting individuals, their families, and the wider society. The restricted number of studies on ASD are a limitation to the generalization of these findings Implications for research and practice are discussed, highlighting the importance of screening and acknowledging the possibility of ASD/ADHD in many settings such as psychiatric and forensic.

## Introduction

Neurodevelopmental disorders including Attention Deficit hyperactivity disorder (ADHD), autism (ASD), dyspraxia and tic disorders, affect around 6% to 8% of the adult population (Fayyad et al., 2017; Gillberg & Soderstrom, 2003; Lai et al., 2014). They are associated with significant long-term effects and impair many cognitive and behavioral functions (Charman et al., 2011; Rubia, 2018; Stoodley, 2016). The pathway to care for neurodevelopmental disorders is not always straightforward. In the UK for example, commissioning decisions on services vary across the country (Hurt et al., 2019; Wright et al., 2015) and neurodevelopmental disorders are currently underdiagnosed in the UK (Ginsberg, Quintero et al., 2014; Hayes et al., 2018). The most common neurodevelopmental conditions, ADHD and ASD, affect 5% and 1% of the adult population respectively (Brugha et al., 2016; McLeod et al., 2007). ASD is a lifelong condition resulting in difficulties in social and communication skills, adapting to change, restricted interests, and sensory hypersensitivity (APA, 2013). ADHD is categorized by symptoms of impulsivity, hyperactivity and inattention and can lead to considerable daily impairment, affecting social behavior, schoolwork and family life (Danckaerts et al., 2010; Faraone et al., 2021). It is estimated that millions of adults and children with ASD and/or ADHD are currently undiagnosed in the UK (Hertz-Picciotto & Delwiche, 2009; Lai & Baron-Cohen, 2015; Lamberg, 2003) and this issue prevails around the world. In Denmark for instance, over half of the children surveyed in a national birth cohort who reported ADHD behavior at age 7 were not diagnosed at follow up many years later (Madsen et al., 2018).

Individuals with ASD and/or ADHD (henceforth ASD/ADHD) report difficulties in obtaining appropriate support, diagnosis, and treatment for difficulties associated with their conditions (Camm-Crosbie et al., 2019; Hayes et al., 2018; Sayal et al., 2018). This leads to poorer outcomes for the individual and their families, such as significant academic underachievement and educational problems (Arnold et al., 2020; Barry et al., 2002; Estes et al., 2011), increased prevalence of depression and anxiety (Howlin & Magiati, 2017; Stewart et al., 2006), higher rates of offending behavior and imprisonment (Haskins & Silva, 2006; Robertson & McGillivray, 2015; Young & Thome, 2011), divorce (Anastopoulos et al., 2009), driving accidents (Daly et al., 2014; Groom et al., 2015; Ulzen et al., 2018), unemployment (Halleland et al., 2019; Hedley et al., 2017; Howlin & Magiati, 2017), suicidal thoughts and behaviors (Cassidy et al., 2022; James et al., 2004) and other mental health issues (Able et al., 2007; Ómarsdóttir et al., 2021). Undiagnosed adults with ADHD are more likely to present with difficulties at work (Asherson et al., 2012), substance abuse (Asherson et al., 2012; Folgar et al., 2018) or increased medical incidents and injuries (Asherson et al., 2012; Kittel-Schneider et al., 2019; Swensen et al., 2004). In the case of substance abuse, it has been hypothesized that individuals with undiagnosed or untreated ADHD may use illicit psychostimulants as a form of self-medication (Gudjonsson et al., 2012; Wilens et al., 2007). Undiagnosed parental ADHD can also strongly impact quality of parenting and increase chaotic home life (Chronis-Tuscano et al., 2008). Undiagnosed ASD in adults has been linked to higher rates of psychiatric conditions (C. Nylander et al., 2018), social problems (Bishop-Fitzpatrick et al., 2018), increased vulnerability to sexual abuse in women (Bargiela et al., 2016), chronic pain (Bursch et al., 2004), and suicidal behaviors (Richards et al., 2019).

These considerable risks are attenuated when ASD/ADHD are diagnosed, as diagnosis facilitates access to care or support (DuPaul et al., 2011). For instance, Swedish population-based data suggest that drug treatment of ADHD may reduce criminality, serious traffic accidents, and suicide rates (Chang et al., 2012; Lichtenstein et al., 2012). Hence, early diagnosis and intervention are of utmost importance in improving some of the long-term outcomes for adults living with these conditions.

There are multiple reasons why many children and adults are still undiagnosed. Socio-economic factors often play a role in recognition of ASD/ADHD. The gender perception gap in how these conditions are expressed and impact males and females for instance can bias recognition, as girls are often missed (Lai & Baron-Cohen, 2015; McCrossin, 2022; Quinn & Wigal, 2004; Russell et al., 2011). Additionally, cultural biases also impact the diagnostic process with people from ethnic minority groups (Coker et al., 2016; Coll et al., 1996) and individuals from lower socio-economic backgrounds often missed (Guthrie et al., 2019; Hodgkinson et al., 2017). Diagnosis may also fail to be given due to favorable personal or environmental conditions (e.g., high child IQ, compliant behavior or learned coping strategies) which might mitigate the impairments of the disorders (Ratey et al., 1992). Additionally, lack of training on these conditions and the presence of misconceptions and skepticism from healthcare professionals and teachers often creates barriers in accessing care (French et al., 2019; Malik-Soni et al., 2022; Olety, 2012; Senarath, 2019). A lack of clear understanding of ASD/ADHD and the importance of receiving a diagnosis and treatment exists amongst parents, teachers, healthcare providers (Hamed et al., 2015) as well as negative attitudes of the community (Bisset et al., 2022; Dickter et al., 2020). Strong partnership between these members of the community could start improving access to care (Hamed et al., 2015). Health services for adults with ASD/ADHD also remain scarce and underfunded, often failing to provide support (Camm-Crosbie et al., 2019; Frith & Mira, 1992; Murphy et al., 2016). Additionally, ASD/ADHD may be mistaken for other mental health conditions or are missed in the presence of comorbid disorders (Au-Yeung et al., 2019; Ginsberg, Beusterien et al., 2014; Haskins & Silva, 2006). Finally, lack of diagnosis may be more prevalent in adulthood. Some individuals with ASD/ADHD may not display symptoms of impairment until later in life when environmental circumstances change, particularly if they had moderate symptoms and adequate social/family supports earlier in life. Therefore, although symptoms might have existed during childhood, impairment might only appear for the first time during adulthood (Lewis, 2018). Moreover, adults may have acquired adaptive behaviors or strategies that help them mask symptoms and impairments in day-to-day settings or make considerable adjustments at great personal cost to mitigate the impact of their symptoms (Bradley et al., 2021; Kosaka et al., 2019; Lewis, 2018), making ASD/ADHD harder to spot.

While there is a wealth of knowledge regarding how ASD/ADHD impacts the lives of individuals, little is known about the traits of adults with ASD/ADHD who are undiagnosed or about the human costs incurred by the disorder among these people. This systematic review identifies and synthesizes the risks associated with late/undiagnosed ASD/ADHD reported in the published literature. No synthesis of these risks has been conducted to date and these novel findings may help to inform future research and policy development.

## Methods

This review was written in accordance with the Preferred Reporting Items for Systematic Reviews and Meta-analysis Protocols (PRISMA-P) guidelines (Moher et al., 2015). A protocol for this review is registered with the International Prospective Register of Systematic Reviews (PROSPERO;355458).

### Search Strategy

Four databases (PsycInfo, Embase, Scopus, Medline) were searched. PROSPERO was checked for ongoing or already published systematic reviews on the subject. A full search strategy for Medline (MEDLINE In-Process & Non-Indexed Citations and OVID MEDLINE 1946 to present-Ovid) is detailed in Supplemental Material as an example and included terms such as: Attention Deficit Disorder with Hyperactivity/(ADHD or ADDH or “attention deficit disorder*” or “attention deficit hyperactivity disorder*” or “hyperkinetic disorder*”), exp Autism Spectrum Disorder/(autis* or ASD or ASC or asperger*). Adaptation of the MEDLINE search strategies was made for the other databases according to their own search criteria. The search was performed on the 5th of December 2022, date limits were not imposed. While hand searching was not a strong component of our planned search strategy, the reference lists of all papers that meet the inclusion criteria were hand searched to check for any additional studies.

### Inclusion Criteria

#### Type of Studies

Quantitative and qualitative studies were included. The qualitative component of this review considered qualitative studies of any design exploring the impacts, effects or risks associated with undiagnosed ADHD/ASD (including, but not limited to ethnography, phenomenology and grounded theory studies). The quantitative component of this review included quantitative studies of experimental and observational designs (including, but not limited to cohort studies, cross-sectional surveys, Randomized controlled trials). Mixed method studies were also included, relevant qualitative and quantitative components extracted separately. Studies published in peer-reviewed publications were solely considered.

#### Type of Population

This review covers studies examining individuals (adults and children) who have not yet received a diagnosis of ASD/ADHD but show traits/symptoms of these disorders on validated screening and assessment tools (such as the adult ADHD self report scale—ASRS or Autism Diagnostic Observation Schedule—ADOS). If studies included multiple groups such as diagnosed and undiagnosed individuals, undiagnosed findings were extracted and reported separately. Studies where individuals are on the care pathway to get a diagnosis as well as studies looking into untreated/unsupported ADHD/ASD were included if the reason for lack of treatment was due to lack of diagnosis.

#### Type of Phenomenon of Interest

This review is examining the impact of not receiving a diagnosis of ADHD/ASD when symptoms of ASD/ADHD are present and meet diagnostic or screening criteria. Within the context of this study, impacts are defined as any consequence from having these conditions in daily life and include consequences to the individuals, their environment (such as job, schools, friendships), their families and any others impacted. This definition and concept of impact vary between each study therefore this review looked at broader concepts such as risks, effects, or consequences. This review considered studies focusing on ADHD/ASD throughout the lifespan and therefore included studies of children, adolescents, and adults.

#### Context

This review was conducted in any setting and took an international perspective. The period of the review was not restricted, covering all publications from inception up to the 5th of December 2022.

### Exclusion Criteria

Unpublished and gray literature were excluded. Studies were also excluded if they did not specify the status of diagnosis examined or focusing on diagnosed individuals and studies that do not mention the terms “Attention Deficit Hyperactivity Disorder,” “Autistic,” “ADHD,” “Autism” or “ASD.” Case studies and studies on genetics, medication and scale development were excluded as well as studies not published in English. Studies that did not separate findings from undiagnosed and diagnosed groups were also excluded.

### Study Selection

**Figure 1.**
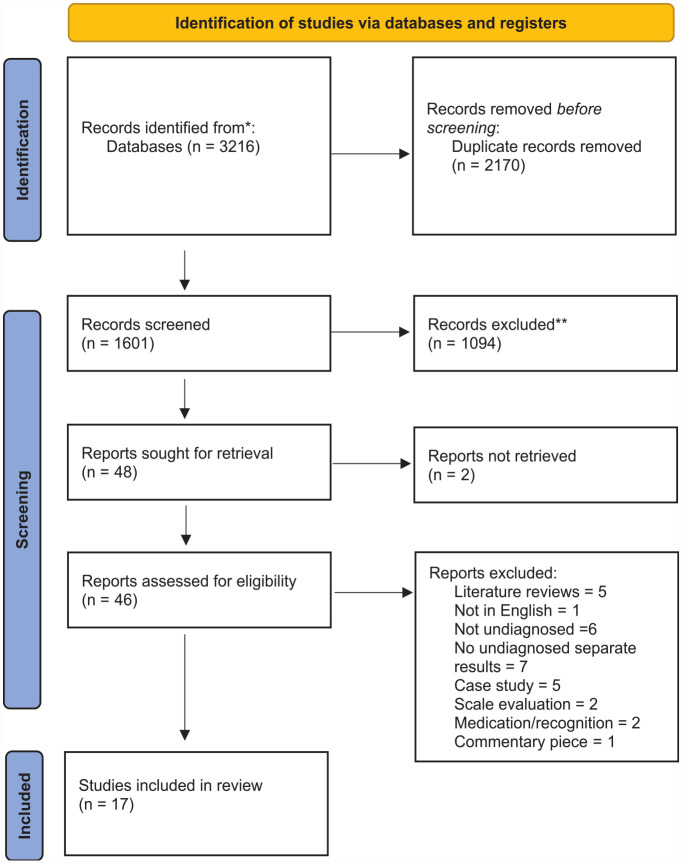
Study selection flow diagram. *Consider, if feasible to do so, reporting the number of records identified from each database or register searched (rather than the total number across all databases/registers). **If automation tools were used, indicate how many records were excluded by a human and how many were excluded by automation tools. *Source.* Page et al. (2021). For more information, visit: http://www.prisma-statement.org/.

Following the search, all identified citations were uploaded into a reference manager software (Zotero). Two of the review authors independently screened the titles and abstracts for assessment against the search inclusion criteria (BF and DD). Full reports were obtained for all titles that appear to meet the inclusion criteria. The same two review authors screened and assessed the full text reports in detail against the inclusion criteria. Disagreement on selected studies were resolved through discussion and/or presented to a third reviewer (SC). Studies that do not meet the inclusion criteria were excluded and a record of reasons for excluding studies can be found in Supplemental Material S1. The study selection process is presented in Figure 1.

### Data Extraction and Outcomes

#### Data Extraction

The lead reviewer (BF) extracted qualitative and quantitative data from the included studies using a standardized data extraction tool (Peters et al., 2015). The data extracted included specific details about the criteria detailed above, aiming to answer the review’s primary objective.

#### Outcomes

The main outcome is the synthesis of risks associated with having undiagnosed ADHD/ASD. Multiple types of factors reported in the selected studies were evaluated such as societal factors (divorce, imprisonment etc.) and health factors (suicide, drug abuse, etc.). These factors were grouped into themes within the synthesis phase.

### Data Synthesis

Due to the mixed-method nature of this review, a segregated synthesis is proposed where two distinct analyses of qualitative and quantitative evidence were made prior to conducting a mixed-methods synthesis (Sandelowski et al., 2013).

A meta-synthesis summarized the qualitative findings. This aggregation or synthesis of findings generated a set of statements representing the aggregation and categorizing them based on similarity in meaning. The findings were presented in narrative form. As statistical pooling was not possible for quantitative data, the findings were presented in a narrative form including tables and figures. The two analyses were then aggregated and combined in the form of qualitative themes (Pearson et al., 2015). Two reviewers conducted the syntheses in sequential order, one reviewer developing the synthesis (BF) and the second checking the findings (DD).

### Assessment of Methodological Quality

Following mixed methods reviews guidelines (Pearson et al., 2015), the process was separated between qualitative and quantitative studies. Two review authors (BF and DD) critically appraised all selected studies for methodological quality using standardized quality appraisal tool (JBI tool Porritt et al., 2014). Any disagreement between reviewers was resolved through discussion and/or a third reviewer (SC). The results are reported in the main study table (Table 1). Study quality did not affect inclusion within the report with all studies going through the data extraction and synthesis process.

**Table 1.** Included Studies Characteristics.

Reference	Country	Population of interest and sample size	Method used for diagnosis/screening	Quality rating	Synopsis of findings	Themes
*Autism*						
Aggarwal and Angus (2015)	Australia	Young people aged 15–25 (*n* = 31 undiagnosed)	ASD assessment: Autism Diagnostic Observation Schedule (ADOS), interviews with parents, review by psychiatrist and two clinicians	High	Adolescents getting referred for an ASD diagnosis presented with: Depression Anxiety Primary psychotic symptoms	Health
Hill et al. (2021)	United States	Children aged 8 (*n* = 243 newly diagnosed)	Surveillance data including screening and abstracting health and/or education records and a systematic review of all records by trained clinicians.	High	Children newly diagnosed compared to already diagnosed children with autism Children newly diagnosed were: More likely to be female More likely to be aggressive and argumentative Less likely to have presented with difficulties at an early age	Offending behavior
Stagg and Belcher (2019)	United Kingdom	Older adults aged 52–54 (*n* = 9 newly diagnosed)	Interviews conducted post diagnosis	High	Interviews conducted with older adults who had recently received a diagnosis Participants reported always having been different and having social and relationship concerns	Day to Day impact
*ADHD*						
Able et al. (2007)	United States	Adults (*n* = 752 undiagnosed)	Screening through telephone survey, using the Adult ADHD Self Report Scale (ASRS)	High	Comparison between an undiagnosed, diagnosed and control group. The undiagnosed group demonstrated higher rates of: Depression Drinking Lower educational attainment Greater emotional and interpersonal difficulties. Lower quality of life	Health Offending behavior Day to Day impact
Bitter et al. (2019)	Czech republic and Hungary	Psychiatric adults inpatients (*n* = 708 undiagnosed)	Screening through ASRS plus clinical interviews	High	The study aimed to estimate the prevalence of undiagnosed ADHD in adults in psychiatric services 9.27% of patients were undiagnosed with ADHD and met diagnostic criteria As well as having psychiatric disorders, this group was more likely to commit suicide	Health
Buitelaar and Ferdinand (2016)	Netherlands	Criminal male adults aged 18 to 51 (*n* = 59 undiagnosed)	DSM IV self-report questionnaire plus clinical interview. Questionnaire was completed by patients and parents/partners	High	The study aimed to assess if adults with ADHD in a forensic sample had received a diagnosis 59 out of 106 adults had ADHD but had not received a diagnosis A high proportion of criminal adults have undiagnosed ADHD Adults newly diagnosed were More likely to be older More likely to have an inattentive subtype More likely to have received care for mental health	Health Offending behavior
Huntley et al. (2012)	United Kingdom	Alcohol and drug adult inpatients (*n* = 226)	DSM IV self-report questionnaire plus clinical interviews	High	12% of substance used disorder patients had undiagnosed ADHD Those individuals had Higher impairments across several domains of daily life (work, money management, education, social interactions, driving and relationships) Higher rates of substance abuse and alcohol consumption Higher rates of suicide attempts and depression	Health Offending behavior Day to Day impact
Kaye et al. (2013)	Australia	Illicit psychostimulant adult users (*n* = 269)	Screening through ASRS and structured interviews	Moderate	This study aimed to estimate the prevalence of undiagnosed ADHD amongst drug users 35% of the participants screened positive for adult ADHD but had not received a diagnosis. This group had: Earlier initiation of substance use Higher frequency of substance use Greater likelihood of dependence and of having received previous treatment Fewer years of education Earlier initiation of tobacco use Higher rates of mental health diagnosis	Offending behavior Day to Day impact
Kumar et al. (2018)	India	Alcohol dependant male adults aged 18–60 (*n* = 50)	Screening through ASRS diagnosis was confirmed by (DSM-5) criteria for adult ADHD and confirmed cases were subjected to ADHD-Rating Scale-IV (ADHD-RS-IV) to assess the severity of symptoms.	High	62% of the group met diagnostic criteria for ADHD High rate of alcohol misuse in undiagnosed ADHD The group also had: Higher chances of smoking Fewer years of education	Offending behavior Day to Day impact
Kuzmickaitė et al. (2019)	Lithuania	Young adult male prisoners (*n* = 100)	Screening through ASRS and the Wender Utah Rating Scale (WURS).	High	17% of the prisoners met ADHD diagnosis criteria and had not received a previous diagnosis This group had: More substance misuse More psychiatric diagnoses and psychopharmacological treatments Higher sleep disorder Higher phobic anxiety	Health Offending behavior
Levin et al. (1998)	United states	Cocaine adult users (*n* = 281)	Interviews with two trained interviewers. PRS was used, a modification of the Conners’ Teacher’s Rating Scale and Abbreviated Scale	Moderate	12% of the sample met diagnostic criteria for ADHD without previous diagnosis Aside for regularly misusing drugs, those participants were more likely to have: A history of conduct and antisocial disorder Fewer years of education Lower yearly income	Offending behavior
Luderer et al. (2020)	Germany	Alcohol-dependant adults (*n* = 415)	Diagnostic Interview for ADHD in Adults (DIVA) conducted by two medical doctors	High	20.5% of inpatients with alcohol dependence met ADHD diagnostic criteria and had not received a diagnosis This groups was: More likely to use illicit substances More likely to have earlier alcohol dependence and more severe	Offending behavior Day to Day impact
Maxson et al. (2009)	United States	Children admitted to hospital aged 6–12 (*n* = 200)	Screening through the NICHQ Vanderbilt Attention-Deficit/Hyperactivity Disorder Parent Rating Scale (VADPRS)	High	The study looked at the prevalence of ADHD between two groups of children admitted to hospital. One with specific injury mechanisms and one with appendicitis. The injured patient group was 3.25 times more likely to screen positive for ADHD Among the patients who screened positive for ADHD 66% had not received a diagnosis	Health
McAweeney et al. (2010)	United States	Adults with substance abuse (*n* = 87)	Screening through ASRS	High	43.68% of adults admitted to a public funded 28-day residential treatment program screened positive for ADHD without previous diagnosis	Offending behavior
Naya et al. (2021)	Japan	Adults (*n* = 9,643)	Screening through ASRS	High	Of the total sample 539 screened positive for ADHD but had not received a diagnosis This group was: Less likely to be married Less likely to have completed university Lower household income More absenteeism at work and more activity impairment at work More hospitalizations and doctors visits More likely to be alcoholics The undiagnosed group reported: Higher coexistence of mental comorbidities More sleep problems More physical comorbidities	Health Offending behavior Day to Day impact
Okumura et al. (2021)	Japan	Children aged 10–12 (*n* = 2,945)	ADHD symptoms were assessed using the parent-rated five-item Strengths and Difficulties Questionnaire (SDQ)-hyperactivity/inattention subscale	High	Of the sample 91 children screened positive for ADHD and had not received a diagnosis The presence of undiagnosed ADHD was significantly associated with: Worse psychosocial functioning Lower self-esteem Higher depression Higher emotional symptoms and conduct problems Higher peer relationship problems Higher risk of self-harm	Health Offending behavior Day to Day impact
Wood et al. (2021)	United states	Young adults college students (*n* = 38 undiagnosed)	Screening through the Barkley Adult ADHD Rating Scale–IV (BAARS-IV)	High	This study compared the psychological profile of students with an ADHD diagnosis, control without ADHD and students without a diagnosis with above-threshold AHD symptoms The group of undiagnosed ADHD but with high traits had: Higher rates of anxiety and depression Higher rates of impairments and procrastination	Health Day to Day impact

## Results

The studies included in this review give an international view of the topic discussed with studies from nine different countries (six from the United states (Able et al., 2007; Hill et al., 2021; Levin et al., 1998; Maxson et al., 2009; McAweeney et al., 2010; Wood et al., 2021), two each from Australia (Aggarwal & Angus, 2015; Kaye et al., 2013), United Kingdom (Huntley et al., 2012; Stagg & Belcher, 2019) and Japan (Naya et al., 2021; Okumura et al., 2021) and one each from Czech Republic and Hungary (Bitter et al., 2019), the Netherlands (Buitelaar & Ferdinand, 2016), India (Kumar et al., 2018), Lithuania (Kuzmickaitė et al., 2019) and Germany (Luderer et al., 2020). Three studies looked at the risks associated with having undiagnosed ASD (Aggarwal & Angus, 2015; Hill et al., 2021; Stagg & Belcher, 2019) while 14 focused on undiagnosed ADHD. The studies explored a wide range of populations: three studies looked at undiagnosed children, two at young adults and students, three at the general adult population while nine looked at a specific adult population (for instance adults with alcohol dependence, or adults in prison).

The findings were synthesized with three main themes of risks associated with undiagnosed ASD/ADHD. The results are presented for ADHD, first, by focusing on the three themes and, then, for ASD, in a single paragraph, due to limited number of studies retrieved.

- Physical and mental Health
- Offending behavior
- Day-to-day impact

### Physical and Mental Health

Undiagnosed ASD/ADHD was most strongly linked to health concerns, principally mental health but also physical health.

#### Mental Health and Wellbeing

Undiagnosed ADHD strongly impacts mental wellbeing with over half of the 17 studies, mentioning a link to poor mental health. Nine studies highlighted the impact of undiagnosed ADHD on long term mental health (Able et al., 2007; Bitter et al., 2019; Buitelaar & Ferdinand, 2016; Huntley et al., 2012; Kaye et al., 2013; Kuzmickaitė et al., 2019; Naya et al., 2021; Okumura et al., 2021; Wood et al., 2021). The studies demonstrated that in adults, undiagnosed ADHD led to higher rates of depression (Able et al., 2007; Huntley et al., 2012; Wood et al., 2021), lower rates of quality of life (Able et al., 2007), higher rates of suicide attempts (Huntley et al., 2012), greater emotional difficulties (Able et al., 2007) and anxiety (Kuzmickaitė et al., 2019; Wood et al., 2021). For instance, in a study of psychiatric inpatients, almost 10% of the patients had undiagnosed ADHD; twice the rate in the general population (Bitter et al., 2019). These patients were also more likely to use psychiatric services, have a psychiatric diagnosis, and were more likely to die by suicide. Adults with undiagnosed ADHD were more likely to have received care for mental health (Buitelaar & Ferdinand, 2016; Kaye et al., 2013) as well as more likely to be diagnosed and treated for comorbid psychiatric diagnoses (Kuzmickaitė et al., 2019; Naya et al., 2021). These findings also translate to children. In a sample of 10 to 12 years old, Okumura et al. (2021) demonstrated that undiagnosed ADHD was significantly associated with lower self-esteem, higher depression, higher emotional symptoms and higher risks of self-harm.

#### Physical Health

Three studies demonstrated a link between undiagnosed ADHD and physical health (Kuzmickaitė et al., 2019; Maxson et al., 2009; Naya et al., 2021). Kuzmickaitė et al. (2019) showed that a group of young male prisoners with undiagnosed ADHD had higher rates of sleep disorders. Similarly, Naya et al. (2021) found that adults with undiagnosed ADHD had more sleep problems, more hospitalizations and doctors’ visits, and more physical comorbidities. In a study of children admitted to emergency services for injuries, versus children admitted for appendicitis, the injured group was over three times more likely to have undiagnosed ADHD (Maxson et al., 2009).

### Offending Behavior

Offending behavior was a common theme associated with undiagnosed ADHD, including imprisonment, crimes, substance abuse and antisocial behavior.

#### Substance Abuse

Substance abuse is one of the main reported impacts of undiagnosed ADHD with nine studies highlighting this link (Able et al., 2007; Huntley et al., 2012; Kaye et al., 2013; Kumar et al., 2018; Kuzmickaitė et al., 2019; Levin et al., 1998; Luderer et al., 2020; McAweeney et al., 2010; Naya et al., 2021), including alcohol, drug and tobacco use. Undiagnosed ADHD was strongly related to alcohol problems (Able et al., 2007; Huntley et al., 2012; Kumar et al., 2018; Luderer et al., 2020; Naya et al., 2021). Luderer et al. (2020) showed that 20% of inpatients with alcohol dependence met ADHD diagnostic criteria and had never received a diagnosis. This group was also more likely to use illicit substances and have earlier and more severe alcohol dependence. Drug abuse was also strongly prevalent (Huntley et al., 2012; Kaye et al., 2013; Kuzmickaitė et al., 2019; Levin et al., 1998; Luderer et al., 2020; McAweeney et al., 2010). McAweeney et al. (2010) found that 43% of adults admitted to a residential substance abuse program had undiagnosed ADHD. In an Australian sample of illicit psychostimulant users, 35% of participants screened positive for ADHD but had not received a diagnosis (Kaye et al., 2013). This group also had earlier initiation and higher frequency of substance use as well as greater likelihood of dependence. Tobacco was also linked to undiagnosed ADHD (Kumar et al., 2018), with earlier initiation of tobacco use compared with control and diagnosed group (Kaye et al., 2013).

#### Crime, Prison, and Antisocial Behavior

Two studies demonstrated a link between undiagnosed ADHD and criminal behavior leading to imprisonment (Buitelaar & Ferdinand, 2016; Kuzmickaitė et al., 2019). In a forensic sample of adult males, over half of the sample of criminal adults had undiagnosed ADHD (Buitelaar & Ferdinand, 2016). In a Lithuanian prison sample of young male adults, 17% of the prisoners met ADHD diagnostic criteria but had not received a diagnosis (Kuzmickaitė et al., 2019). Undiagnosed ADHD was also linked to conduct and antisocial disorder (Levin et al., 1998) and conduct problems (Okumura et al., 2021).

### Day-to-Day Impact

Finally, undiagnosed ADHD had broader impacts on day-to-day activities such as driving, education, work and relationships. Many studies highlighted the impact undiagnosed ADHD had on education and working life with a consistent finding of fewer years of education (Able et al., 2007; Huntley et al., 2012; Kaye et al., 2013; Kumar et al., 2018; Levin et al., 1998; Naya et al., 2021) and lower yearly income (Levin et al., 1998; Naya et al., 2021). Higher rates of functional impairments (such as relationships, self-care, money management, social interactions) and procrastination were also observed (Wood et al., 2021). Naya et al. (2021) showed that adults with undiagnosed ADHD were less likely to have completed University, had lower household income, had more absenteeism at work and were more impaired in work-related activities. Additionally, undiagnosed adults with ADHD had more difficulties with money management and were more prone to careless driving (Huntley et al., 2012). Undiagnosed ADHD led to greater social and relationship difficulties. Higher rate of impairments in social interactions and relationship were observed (Huntley et al., 2012) with greater interpersonal difficulties (Able et al., 2007).

### ASD

Only three studies were identified on ASD. These studies covered topics from the three key themes discussed above and related more specifically to mental health, social difficulties and aggressive behavior.

Similarly to the studies on ADHD, in an ASD referral clinic, adolescents with undiagnosed ASD presented with higher rates of depression, anxiety and psychotic symptoms (Aggarwal & Angus, 2015), the most common presentation being depressive symptoms.

Undiagnosed ASD was also linked to feeling like you are different. A group of late diagnosed older adults explained that living with undiagnosed ASD created social and relationship concerns (Stagg & Belcher, 2019). This group also received treatment for anxiety and depression throughout most of their lives. Finally, a review of health records showed that children with undiagnosed ASD were more likely to be aggressive, argumentative and have behavioral difficulties at an early age (Hill et al., 2021).

## Discussion

This review highlights many impacts and risks associated with ASD/ADHD, impacting the individuals but also healthcare, social, and forensic systems. These risks have been aggregated into three key themes encompassing health related risks, the links to offending behaviors and the impacts on daily activities. The most prominent finding across studies related to the impact of a lack of diagnosis on mental health, substance abuse and education/work. While these themes were linked to both conditions, it is important to note that so few studies had been published on undiagnosed ASD that, with only three studies on ASD, we cannot generalize our findings across ASD and this discussion will primarily focus on ADHD.

The health risks related both to mental and physical health. Physical health comorbidities were reported less than mental health and mainly linked to increased visits to healthcare professionals or services and increased injury and accidents. This finding is significant as unintentional injury is the leading cause of mortality among children between the ages of 1 and 14 years in the United States (Runyan et al., 2005). Numerous studies have shown that children and adults with ADHD have a higher frequency of injuries compared to controls (Adeyemo et al., 2014; Brunkhorst-Kanaan et al., 2021) and children are more likely to sustain severe injuries (DiScala et al., 1998). Therefore, it is important to highlight that a significant proportion of children accessing emergency services could be at risk of ADHD, but undiagnosed, and children with a frequent history of accidents or presentation at emergency services could benefit from being screened for ADHD.

The mental health risks linked with ADHD were the most recurrent finding across studies. Even studies not directly looking at mental health outcomes, consistently found negative impacts of lack of diagnosis on mental health (e.g., through medical record screening). The most common mental health reported difficulties were depression and anxiety but also included psychiatric inpatient admissions and suicidality. These mirror previous findings on the impact of lack of diagnosis on mental health (Able et al., 2007; Howlin & Magiati, 2017). In a recent review of an Icelandic ADHD clinic, Ómarsdóttir et al. (2021) found that adults referred to an ADHD clinic more often met diagnostic criteria for dysthymia, agoraphobia and generalized anxiety, and were more likely to be diagnosed with two or more comorbid disorders prior to their ADHD diagnosis. A national American survey also demonstrated that untreated adult ADHD was highly comorbid with many other DSM-IV disorders and substantial impairment (Kessler et al., 2006). Previous studies have also highlighted correlations between suicidal ideations and ADHD symptomology (mainly undiagnosed - Huemer et al., 2016; James et al., 2004). While there was only limited evidence of mental health risks in ASD in the current study, previous studies have also reported a strong link between undiagnosed ASD, suicidality and psychiatric disorders (Cassidy & Rodgers, 2017; L. Nylander & Gillberg, 2001) as well as with depression and anxiety (Stewart et al., 2006). These studies were not included in the review as they did not meet inclusion criteria (the results between undiagnosed and diagnosed not being separated). Mental health clinicians should routinely consider ASD/ADHD in patients experiencing these difficulties.

Offending behaviors including imprisonment, aggression, criminality, and substance abuse were also a key theme from this review. These results reflect findings from many studies which have shown that high rates of prisoners have undiagnosed/untreated ADHD (Appelbaum, 2008; Ginsberg et al., 2013). ADHD is highly prevalent in prison, in comparison to prevalence rates in the general population. A recent meta-analysis of 42 studies indicated a fivefold higher prevalence of ADHD in young forensic populations (30.1%) and adult ADHD in UK prisoners is 10 times higher (26.2%) than the general population (Young et al., 2015). Additionally, untreated ADHD has been associated with poorer social function outcomes, highlighting the importance of treatment (Harpin et al., 2016) Our analyses also highlighted the relationship to substance abuse, with over half of the studies mentioning some form of substance misuse. Substance abuse included alcohol, drug and tobacco abuse. Previous studies have shown that the estimated prevalence of ADHD amongst adults with substance use disorders is over 20% (van Emmerik-van Oortmerssen et al., 2012). In our studies looking at undiagnosed ADHD, the numbers were much higher ranging from 20% (Luderer et al., 2020), 35% (Kaye et al., 2013) to 43% (McAweeney et al., 2010), suggesting that lack of diagnosis may further increase risk of substance abuse amongst those with ADHD symptoms. Plausible explanations for this could be the self-medication of ADHD symptoms with drugs or alcohol, or ADHD-related impairments in social functioning that can cause social marginalization, leading to involvement in more “deviant” behaviors like drug use (Kollins, 2007). Kaye et al. (2013) demonstrated that the strongest predictors of substance abuse in those undiagnosed were an earlier onset of tobacco use and a greater extent of past polydrug use. ADHD in adults with substance use disorder has been shown to have a negative impact on treatment efficacy and treatment retention (Wilens & Morrison, 2011) and is associated with a more severe course of substance use (Moura et al., 2013), highlighting the importance of having an ADHD diagnosis in substance abuse treatment. Additionally, long-term treatment of ADHD medication such as methylphenidate has been shown to reduce risks of substance misuse (Krinzinger et al., 2019). Routinely considering undiagnosed ADHD in substance abuse clinics as well as prison healthcare settings is therefore of utmost importance.

The day-to-day impact of undiagnosed ASD/ADHD included many risks across different settings. These encompassed social difficulties and difficulties in relationships, difficulties with money, education and work as well as driving. Undiagnosed ADHD was consistently linked to lower income and lower educational attainment. While this impact was not researched directly in any of the studies, six studies demonstrated this as a key additional finding through demographic questionnaires. While these findings mirror current knowledge on the relationship between ADHD and lower educational outcomes (Daley & Birchwood, 2010; Gordon & Fabiano, 2019), our review adds that in a comparison between groups of adults who were either undiagnosed, diagnosed or whose symptoms were controlled by treatment, educational outcomes were worse for the undiagnosed group than the other two groups (Able et al., 2007). Early diagnosis is of utmost importance for improving educational outcomes, most specifically as treated ADHD shows better academic outcomes in comparison to untreated ADHD (Arnold et al., 2020). The impact of lack of diagnosis on relationships and interpersonal difficulties was demonstrated for both ASD/ADHD groups. The feeling of “being an alien, being different” (Able et al., 2007; Stagg & Belcher, 2019) echoes many studies with implications for psychological and psychosocial functioning (Okumura et al., 2021). Peer relationships are often difficult for children and adults with ASD/ADHD (Gardner & Gerdes, 2015; Wood-Downie et al., 2021) and many learn to mask and develop strategies to facilitate social interactions (Miller et al., 2021; Young, 2005). Receiving a diagnosis in adulthood carries many positive consequences, in part being able to explain this feeling of ”being different” (Hansson Halleröd et al., 2015). Our findings support previous studies (Harpin et al., 2016) which have shown that a lack of diagnosis creates worse social impairment for ASD/ADHD individuals. Finally, our results highlights the relationship between ADHD and driving. ADHD has been linked to more risky driving behavior (Thompson et al., 2007) and more driving accidents (Fischer et al., 2007), and our study demonstrates the impact of this when ADHD remains undiagnosed.

This review demonstrates that the risks associated with undiagnosed ASD/ADHD are significant, greatly impairing and need to be taken seriously. While the rates of ASD/ADHD diagnosis has increased in the past decade (Kooij et al., 2019; Russell et al., 2022) and the awareness of these conditions has changed and improved, many millions of individuals remain undiagnosed. Many studies have highlighted the benefits of early diagnosis and early treatment in reducing some of the mental health harms from undiagnosed ASD/ADHD (Koegel et al., 2014; Krinzinger et al., 2019), reinforcing the importance of early identification. This review highlights how damaging this lack of diagnosis can be and the impact it has on many aspects of life, principally on mental wellbeing and the likelihood of substance abuse.

### Strength and Limitations

This review contains some strength and limitations. To the authors’ knowledge, this is the only review that has attempted to establish the specific risks and impacts arising from a lack of ASD/ADHD diagnosis. This review included all studies looking specifically at this topic, regardless of age group and the limited number of included studies suggest that this is an under-researched or difficult to study topic. The main limitation of this review is the small number of studies on ASD. Despite the similarity of topics between the ASD studies and the ADHD studies, with only three included studies on ASD, it is not possible to generalize or indeed differentiate the findings across disorders. While most studies conducted a thorough assessment of ASD/ADHD as part of their methodology (including but not limited to interviews and questionnaires), a few relied solely on screening questionnaires. While these may give a strong inclination of individuals having ASD/ADHD, they are limiting as no official diagnosis process was performed. Similarly, our search criteria aimed to specifically identify groups of “undetected,” “undiagnosed” and “unrecognized” ASD/ADHD. Studies which might have used different terminology would not have been capture by our search or identified in our review of titles, abstract or keywords. This was intentional to limit the number of non-relevant studies or studies where undiagnosed ASD/ADHD was not a key focus, however this may explain the limited number of ASD relevant studies, as many studies tended to assess autistic traits across a whole population, rather than identify a specific group of undiagnosed autistic people. Additionally, a few studies on undiagnosed ASD were excluded due to a lack of separate report of the findings which prevented us to draw conclusions on this population.

### Recommendations

A few recommendations can be made from this review, both on the clinical and research impacts of the findings. Understanding the psychosocial burden of undiagnosed ASD/ADHD will help teachers, clinicians, and policy makers pay more attention to the consequences of under-diagnosis of these conditions. Due to the high rate of undiagnosed ADHD in specific healthcare services such as emergency services (for accidents and injuries), psychiatric services (for mental health related risks) and forensic services (for substance misuse and prisons), it would be advisable that healthcare professionals in these settings regularly screen for potential ADHD, using standardized screening measures such as the adult ADHD self-report scale (ASRS- Kessler et al., 2005) or the Conners’ adult ADHD rating scale (CAARS-Conners et al., 1999). ASD/ADHD presented with strong relationships with mental health issues such as depression and anxiety and clinicians working in psychiatric settings should routinely consider screening for these conditions in children and adults regardless of the reason for referral. In cases of no childhood ASD/ADHD identification, clinicians should carefully assess impairment, psychiatric history, and substance use before treating potential adults with ADHD. Future research should also focus more on understanding the impacts of undiagnosed ASD/ADHD. With only three studies on ASD and fourteen on ADHD, this topic is highly under-researched. More specifically, as many ASD studies historically have looked at traits of ASD in the population, we suggest that future ASD research should look more specifically at undiagnosed ASD to address the lack of knowledge around the risks associated with it. As we have established the worsen impacts of lack of diagnosis, more research should focus on studying this particular group.

## Supplemental Material

sj-rtf-1-jad-10.1177_10870547231176862 – Supplemental material for Risks Associated With Undiagnosed ADHD and/or Autism: A Mixed-Method Systematic Review Click here for additional data file. Supplemental material, sj-rtf-1-jad-10.1177_10870547231176862 for Risks Associated With Undiagnosed ADHD and/or Autism: A Mixed-Method Systematic Review by Blandine French, David Daley, Madeleine Groom and Sarah Cassidy in Journal of Attention Disorders
