# Supplementary material for: Risks Associated With Undiagnosed ADHD and/or Autism: A Mixed-Method Systematic Review
Source: J Atten Disord. 2023 Jun 21;27(12):1393–410. doi: 10.1177/10870547231176862 (PMC10498662; doi:10.1177/10870547231176862)
Supplement: sj-rtf-1-jad-10.1177_10870547231176862 – Supplemental material for Risks Associated With Undiagnosed ADHD and/or Autism: A Mixed-Method Systematic Review [file sj-rtf-1-jad-10.1177_10870547231176862.rtf]

1.	Asherson P, Akehurst R, Kooij JJS, Huss M, Beusterien K, Sasane R, et al. Under diagnosis of adult ADHD: Cultural influences and societal burden. Able A Adler, Almeida Montes, Antshel, Applegate, Asherson, Asherson, Asherson, Babinski, Barkley, Barkley, Barkley, Barkley, Barkley, Barkley, Bell, Biederman, Biederman, Biederman, Biederman, Birnbaum, Brod, Bukstein, Canino, Chen, Chronis, Clarke, Cohen, Conrad, Cox, Cox, Cox, Davidson, Dias, DuPaul, Eakin, Ebert, Elia, Epstein, Europe, Evans, Eyestone, Faraone, Faraone, Faraone, Feifel, Gervais, Goodman, Hermans, Jerome, Kessler, Kessler, Kessler, Kessler, Kooij, Kooij, Kooij, Lam, Larsson, Lesch, Levy, Mann, Mannuzza, McCarthy, McGough, McLeod, Mehringer, Mick, Milich, Minde, Moncrieff, Moss, Murphy, Oyserman, Polanczyk, Rabiner, Ramsay, Rasmussen, Riccio, Rosler, Rosler, Ryden, Sankaranarayanan, Schmidt, Schmitz, Secnik, Simon, Skirrow, Smith, Sonuga Barke, Sonuga Barke, Stevens, Stevens, Stringaris, Swensen, Taylor, Timimi, Torgersen, Vaa, Wagner, Waite, Wasserstein, Weiss, Weiss, Weiss, Wilens, Williams, Wolraich, Yen, Young, Young, Young, Zucker, editor. Journal of Attention Disorders. 2012;16(5, Suppl):20S-38S. 
Reason for exclusion – Not undiagnosed
2.	De Souza I., Mattos P., Pina C., Fortes D. ADHD: The impact when not diagnosed. J Bras Psiquiatr. 2008;57(2):139–41. 
Reason for exclusion – Case study
3.	Folgar MI, Golpe S, Requeijo MO, Ayesta JA, Salgado PG. Tobacco use and self-esteem in adolescents with and without Attention Deficit Disorder with Hyperactivity (ADHD): Proposals for better prevention. Revista Espanola de Drogodependencias. 2018;43(4):69–82. 
Reason for exclusion – In Spanish
4.	Garnier-Dykstra LM, Pinchevsky GM, Caldeira KM, Vincent KB, Arria AM. Self-reported adult attention-deficit/hyperactivity disorder symptoms among college students. Adler A Arria, Biederman, Bloom, Davidson, Graaf, DuPaul, Fayyad, Gittelman, Heiligenstein, Kessler, Kessler, Mannuzza, Reuter, Weiss, Weyandt, Weyandt, editor. Journal of American College Health. 2010;59(2):133–6. 
Reason for exclusion – Report scale evaluation
5.	Gindlesperger MF. Neuropsychological effects of illicit substance use among individuals with symptoms of attention-deficit/hyperactivity disorder (adhd). Dissertation Abstracts International: Section B: The Sciences and Engineering. 2015;75(12-B(E)):No-Specified. 
Reason for exclusion – Not undiagnosed
6.	Ginsberg Y., Quintero J., Anand E., Casillas M., Upadhyaya H.P. Underdiagnosis of attention-deficit/hyperactivity disorder in adult patients: A review of the literature. Prim Care Companion J Clin Psych [Internet]. 2014;16(3). Available from: http://www.psychiatrist.com/private/pccpdf/article_wrapper.asp?art=2014/13r01600/13r01600.htm
Reason for exclusion – Review
7.	Ginsberg Y, Beusterien KM, Amos K, Jousselin C, Asherson P. The unmet needs of all adults with ADHD are not the same: A focus on Europe. Able A Adamou, Aragones, Asherson, Asherson, Bahmanyar, Barrett, Benkert, Bitter, Bitter, Blix, Bolea, Brassett Grundy, Brod, Bruce, Brummer, Caci, Cadman, Camilleri, Chitsabesan, Coogan, De Graaf, De Zwaan, Edebol, Edvinsson, Edwin, Einarsson, Emilsson, Faraone, Fayyad, Fischer, Fried, Ghanizadeh, Ginsberg, Ginsberg, Ginsberg, Guldberg Kjar, Hall, Halmoy, Hinshaw, Hirvikoski, Johnson, Joy Tong, Kiejna, Knapp, Konstenius, Konstenius, Kooij, Kooij, Kotsopoulos, Kupper, Lichtenstein, Lillemoen, Marcer, Matheson, McCarthy, Mehlkopf, Michielsen, Newark, Nutt, Nylander, Ogundele, Pehlivanidis, Pentek, Philipsen, Philipsen, Pottegard, Ramos Quiroga, Rao, Retz, Rosler, Sandell, Schlander, Sexias, Swift, Syed, Taylor, Toner, van de Glind, van Emmerik van Oortmerssen, Yargic, Young, Young, Young, Young, Young, Zetterqvist, editor. Expert Review of Neurotherapeutics. 2014;14(7):799–812. 
Reason for exclusion – Review
8.	Gupta M, Chaudhary R. Diagnostic Challenges of High-Functioning Autism Spectrum Disorder in Females. Cureus. 2021;13(1):e13006. 
Reason for exclusion – Case study
9.	Haskins BG, Silva JA. Asperger's disorder and criminal behavior: Forensic-psychiatric considerations. Abu-Akel BO Baron Cohen, Baron Cohen, Baron Cohen, Baron Cohen, Baron Cohen, Barry Walsh, Beglinger, Brune, Chakrabarti, Chen, Clarke, Constantino, Cooper, Critchley, Everall, Fombonne, Frith, Frith, Ghazziudin, Gillberg, Gillberg, Grady, Hall, Haracopos, Hare, Hare, Howlin, Khouzam, Kohn, Lord, Lord, Mawson, Meloy, Mullen, Murrie, Ousley, Ozonoff, Palermo, Peterson, Povinelli, Santangelo, Scragg, Shedler, Silva, Silva, Silva, Silva, Silva, Siponmaa, Smith, Stokes, Stone, Sverd, Tantam, Tantam, Tietz, Volkmar, Volkmar, Wing, Wing, Wolff, Wolff, editor. Journal of the American Academy of Psychiatry and the Law. 2006;34(3):374–84. 
Reason for exclusion – Not undiagnosed and case study
10.	Huemer J., Riegler A., Volkl-Kernstock S., Wascher A., Lesch O.M., Walter H., et al. The influence of reported ADHD and substance abuse on suicidal ideation in a non-clinical sample of young men. Neuropsychiatrie. 2016;30(3):131–7. 
Reason for exclusion – No undiagnosed separated results
11.	Knecht C, de Alvaro R, Martinez-Raga J, Balanza-Martinez V, Carabal E. Criminality and attention deficit hyperactivity disorder. Adler A Amsten, Andrade, Arias, August, Bange, Barkley, Barkley, Barkley, Beaver, Beaver, Beaver, Bemat, Bobb, Breuk, Brown, Buitelaar, Burke, Bushe, Bussing, Byrd, Cahill, Carpentier, Carrasco, Chae, Charach, Cho, Cohn, Colins, Colins, Comings, Connor, Coolidge, Coolidge, Crowell, Crowley, Cunill, Daley, Dalsgaard, De Alwis, De Andrade, De Sanctis, De Sanctis, Delisi, Dietrich, Disney, Edvinsson, Einarsson, Ekiund, Elkins, Emilsson, Ercan, Ercan, Evans, Eyestone, Faraone, Faraone, Fazel, Feldman, Fergusson, Fergusson, Fletcher, Folino, Forehand, Gaiffas, Ghanizadeh, Ghanizadeh, Ginsberg, Ginsberg, Gizer, Goldschmidt, Gordon, Gosden, Grieger, Groenman, Gudjonsson, Gudjonsson, Gunter, Guo, Guo, Halikas, Harty, Harzke, Hazell, Hennessey, Henrichs, Humphrey, Impey, Jerome, Johansson, Jones, Kafka, Kim, Kooij, Kooij, Lam, Lambie, Langcvin, Langley, Lee, Lichtenstein, Lie, Lindberg, Lindsay, Loeber, Mannuzza, Mannuzza, Martinez Raga, Matsumoto, McCarthy, Milin, Mill, Miller, Moffitt, Molina, Molina, Molina, Montano, Mordre, Mrug, Mundt, Muris, Oner, Ortiz, Pardini, Piatigorsky, Pineiro Dieguez, Pingault, Plattner, Polanczyk, Posthumus, Ramos Quiroga, Retz, Retz, Rey, Ristow, Romine, Rosier, Rosier, Satterfield, Satterfield, Satterfield, Schilling, Schubert, Sevecke, Shaffer, Sheehan, Sibley, Sibley, Soderstrom, Soltis, Teplin, Thompson, Torok, van Emmerik van Oortmerssen, Vitacco, Von Poller, Vreugdenhil, Wallis, Walther, Washburn, Westmoreland, Wilens, Wu, Yoo, Yoshimasu, Young, Young, Young, editor. Forensic psychiatry: A public health perspective. 2015;(Adler, L. D., Nierenberg, A. A. (2010). Review of medication adherence in children and adults with ADHD. Postgrad Med 2010;122:184-91.):119–38. 
Reason for exclusion – study on medication
12.	Manos MJ. Nuances of Assessment and Treatment of ADHD in Adults: A Guide for Psychologists. Professional Psychology: Research and Practice. 2010;41(6):511–7. 
		Reason for exclusion – Not undiagnosed
13.	Morstedt B, Corbisiero S, Bitto H, Stieglitz RD. Attention-deficit/hyperactivity disorder (ADHD) in adulthood: Concordance and differences between self- and informant perspectives on symptoms and functional impairment. Able A Bagwell, Barkley, Barkley, Barkley, Barkley, Barkley, Barkley, Barkley, Biederman, Biederman, Canu, Canu, Christiansen, Christiansen, Conners, Corbisiero, Danckaerts, de Zwaan, Eakin, Edel, Fossati, Friedman, Glutting, Goodman, Gordon, Harpin, Hoza, Jacobson, Jensen, Kendall, Knouse, Knouse, Kooij, Kooij, Magnusson, Miller, Millstein, Minde, Moeller, Murphy, Newark, Nijmeijer, Owens, Philipsen, Prevatt, Ratey, Retz Junginger, Rizzo, Robbins, Robin, Rosler, Saunders, Sobanski, Taylor, Turgay, Wasserstein, Wender, Wender, Wender, Wolraich, Zucker, editor. PLoS ONE. 2015;10(11). 
Reason for exclusion – Symptom rating scale
14.	Pinkhardt E.H., Kassubek J., Brummer D., Koelch M., Ludolph A.C., Fegert J.M., et al. Intensified testing for attention-deficit hyperactivity disorder (ADHD) in girls should reduce depression and smoking in adult females and the prevalence of ADHD in the longterm. Med Hypotheses. 2009;72(4):409–12. 
Reason for exclusion – Not undiagnosed and review
15.	Prevatt F, Young JL. Recognizing and Treating Attention-Deficit/Hyperactivity Disorder in College Students. Journal of College Student Psychotherapy. 2014;28(3):182–200. 
Reason for exclusion – Study on recognition
16.	Puri M., Bekker Y., Islam F.A. Is your patient using cocaine to self-medicate undiagnosed ADHD? Curr Psychiatry. 2014;13(12):e3–4. 
Reason for exclusion – Commentary piece
17.	Rucklidge JJ, Kaplan BJ. Attributions and perceptions of childhood in women with ADHD symptomatology. Attention-Deficit/Hyperactivity Disorder. J Clin Psychol. 2000;56(6):711–22. 
Reason for exclusion – No undiagnosed separated results
18.	Ulzen TP, Higginbotham JC, Donnir G, Jerome L, Segal A. Undiagnosed attention deficit/hyperactivity disorder (ADHD) among unionized drivers in Ghana: Public health and policy implications. Adler A Barkley, Barkley, Barkley, Barkley, Bukstein, Chang, Cox, Cox, Diener, Faraone, Hasselberg, Jerome, Jerome, Ji Hae, Kooij, Lam, Mannuzza, Polanczyk, Polanczyk, Siaw, Skirrow, Treat, Ulzen, Vaa, Weafer, Wilens, Willcutt, Yeh, Zohar, Zucker, editor. Accident Analysis and Prevention. 2018;114(Transportation [4090]):12–6. 
Reason for exclusion – No undiagnosed separated results
19.	Valkanova V, Rhodes F, Allan CL. Diagnosis and management of autism in adults. Practitioner. 2013;257(1761):13–3. 
Reason for exclusion – Not undiagnosed 
20.	Waite R, Ramsay JR. Adults with ADHD: Who are we missing? Able A Arnold, Arnold, Bailey, Banks, Barkley, Barkley, Biederman, Biederman, Biederman, Biederman, Biederman, Biederman, Bradley, Bussing, Bussings, Bussings, Chronis, Chronis, Chronis, Chronis Tuscano, Collins, Constantine, Cuffe, Cuningham, Dodson, Dwivedi, Elliott, Epstein, Escobar, Faraone, Faraone, Faraone, Garcia Coll, Garcia Coll, Gingerich, Goodman, Guevara, Harvey, Hervey Jumper, Hinnenthal, Katragadda, Koro Ljungberg, Lamberg, Leverton, Mapou, Minde, Molina, Ninowski, Novello, Olaniyan, Pastor, Pauls, Perry, Power, Prince, Prudent, Psychogiou, Quimbly, Quinn, Quinn, Ramsay, Ramsay, Ramsay, Ramsay, Ratey, Ray, Rousseau, Rowland, Rutter, Sonuga Barke, Sonuga Barke, Stevens, Thapar, Todd, Tumulty, Turner, Waite, Waite, Weiss, Weiss, Wells, editor. Issues in Mental Health Nursing. 2010;31(10):670–8. 
Reason for exclusion – Review
21.	Baker DB, Knight K, Simpson DD. Identifying probationers with ADHD-related behaviors in a drug abuse treatment setting. Criminal Justice and Behavior. 1995;22(1):33–43. 
Reason for exclusion – No undiagnosed separated results
22.	Bargiela S, Steward R, Mandy W. The experiences of late-diagnosed women with autism spectrum conditions: An investigation of the female autism phenotype. Allison B Barker, Barker, Constantino, Cridland, Dworzynski, Elliott, Fombonne, Giarelli, Goldberg, Goldberg, Hartley, Head, Hiller, Holdnack, Holliday Willey, Howlin, Huke, Hurlbutt, Kenyon, Kim, Kristensen, Lai, Lai, Makowska, Mandy, Mandy, Mandy, Mays, McLennan, Pellicano, Pope, Portway, Posserud, Ritchie, Ritchie, Ruiz Calzada, Russell, Russell, Rutherford, Rynkiewicz, Sedgewick, Smith, Snaith, Steward, Stiles, Strang, van Wijngaarden Cremers, Wong, Zwaigenbaum, editor. Journal of Autism and Developmental Disorders. 2016;46(10):3281–94. 
Reason for exclusion – Not undiagnosed 
23.	Bursch B, Ingman K, Vitti L, Hyman P, Zeltzer LK. Chronic pain in individuals with previously undiagnosed autistic spectrum disorders. Aftanas B Bauman, Buitelaar, Chugani, Courchesne, Grandin, Grandin, Harlow, Haznedar, Hutt, Kemper, Landa, Mason, Melzack, Nader, Narayan, Schreibman, Simons, Sverd, Tanguay, editor. The Journal of Pain. 2004;5(5):290–5. 
Reason for exclusion – Case study
24.	Carbone MG, Miniati M, Simoncini M, Maglio A, Carmassi C, Dell'Osso L. Undetected autism subthreshold spectrum as risk factor for suicidal gestures in adulthood: A case report. Baron-Cohen BB Chandrasekhar, Dell'Osso, Fombonne, Ghaziuddin, Gillberg, Hedley, Howlin, Kasper, Kato, Mosconi, Munesue, Palermo, Richa, Ritvo, Segers, Storch, Wing, editor. Journal of Psychopathology. 2018;24(1):37–40. 
Reason for exclusion – Case study
25.	Cassidy S., Au-Yeung S., Robertson A., Cogger-Ward H., Richards G., Allison C., et al. Autism and autistic traits in those who died by suicide in England. Br J Psychiatry [Internet]. 2022;((Cassidy, Au-Yeung) School of Psychology, University of Nottingham, Faculty of Health and Life Sciences, Coventry University, United Kingdom). Available 
Reason for exclusion – No undiagnosed separated results
26.	Cheng SH, Lee CT, Chi MH, Sun ZJ, Chen PS, Chang YF, et al. Factors related to self-reported attention deficit among incoming university students. Adams A Atwoli, Bernardi, Biederman, Biederman, Biederman, Carli, Chao, Charach, Chen, Chen, Cheng, Cheng, Cho, Dahlstrom, de Graaf, DuPaul, Fayyad, Garnier Dykstra, Gittelman, Goodman, Gould, Heiligenstein, Impey, Kessler, Kessler, Kessler, Klassen, Ko, Ko, Ko, Lee, Lee, Lee, Lee, Lin, Looby, Montiel Nava, Park, Plattner, Ramos Olazagasti, Sciutto, Semeijn, Tsai, Tsai, Tzang, van Emmerik van Oortmerssen, Verret, Weiss, Weyandt, Wilens, Yao, Yeh, Yeh, Yen, Yen, Yoo, editor. Journal of Attention Disorders. 2016;20(9):754–62. 
Reason for exclusion – No undiagnosed separated results
27.	Hamed AM, Kauer AJ, Stevens HE. Why the diagnosis of attention deficit hyperactivity disorder matters. Able A Atkinson, Bathiche, Batstra, Beard, Bener, Berger, Berger, Bussing, Castle, Chacko, Chen, Comer, Copeland, Correll, Cortese, Dalsgaard, Dalsgaard, Damborsky, de Graaf, Djoubairou, Elkins, Faraone, Faraone, Farrington, Fischer, Fried, Geltman, Ginsberg, Hendriksen, Hervey Jumper, Hinshaw, Huntley, Kiely, Langberg, Loo, McCann, Pastor, Powers, Qu, Rey, Rousseau, Russell, Russell, Saloner, Sankaranarayanan, Santosh, Saul, Sayal, Sayal, Sayal, Schonwald, Secnik, Shattell, Shaw, Short, Sibley, Solanto, Solanto, Sonuga Barke, Stevens, Stevens, Wolraich, Swanson, Taylor, Travell, Vance, Visser, Waite, Weitzman, Winzer Serhan, Young, Zhu, Zuvekas, Zwaanswijk, Zwi, editor. Frontiers in Psychiatry. 2015;6(Able, S. L., Johnston, J. A., Adler, L. A., Swindle, R. W. (2007). Functional and psychosocial impairment in adults with undiagnosed ADHD. Psychol Med, 37, 97-107. http://dx.doi.org/10.1017/S0033291706008713). 
Reason for exclusion – Review
28.	Sanz-Garcia O., Duenas R.M., Domenech N., Ramon R., Muro A., Perez F. How do we treat patients with undiagnosed attention deficit hyperactivity disorder? Psychiatric population study in prison. Eur Neuropsychopharmacol. 2010;20(SUPPL. 3):S623. 
29.	Waite R, Buchanan T, Leahy M. Assessing ADHD Symptoms among Young Adults in the University with the ASRS v1.1: Examining Associations with Social Anxiety and Self-Efficacy. International Journal of Disability, Development and Education [Internet]. 2020; Available from: https://www.scopus.com/inward/record.uri?eid=2-s2.0-85092260992&doi=10.1080%2f1034912X.2020.1825643&partnerID=40&md5=1482705105c9c9496d987c4aa33efae8
Reason for exclusion – No undiagnosed separated results
